# Causes of Suicide

**Published:** 1878-10

**Authors:** 


Causes of Suicide.—Of the 5,567 cases of suicide reported in France during 1876, among the causes assigned are drunkenness, 1,443, afflicted with incurable diseases, 798, domestic broils, 633, dread of poverty, 329, less than one-third of the entire list is charged with drunkenness. —*New York City Hospital Journal.*

